# Isolated Acute Tubulointerstitial Nephritis in a Myeloperoxidase-ANCA-Positive Patient Without Crescentic Glomerulonephritis

**DOI:** 10.7759/cureus.116328

**Published:** 2026-09-15

**Authors:** Vishal Parmar, Ruchir B Dave, Ayman Vakil, Vishal Prajapati, Rumita Padghan, Pranjal Kashiv

**Affiliations:** 1 Nephrology, Institute of Kidney Diseases and Research Centre (IKDRC), Ahmedabad, IND; 2 Medicine, Aartham Hospitals LLP, Ahmedabad, IND; 3 Nephrology, All India Institute of Medical Sciences, Nagpur, IND

**Keywords:** acute kidney injury, acute tubulointerstitial nephritis, anca-associated vasculitis, mpo-anca, pauci-immune vasculitis, renal biopsy

## Abstract

Kidney involvement in ANCA-associated vasculitis is usually recognized by its hallmark lesion, necrotizing crescentic glomerulonephritis, and clinicians naturally anchor their diagnostic expectations to this pattern. Rarely, however, the injury appears largely confined to the tubulointerstitium, with glomeruli that show no necrotizing or crescentic lesions, a presentation that is easily mistaken for drug-induced or infection-related interstitial nephritis. We describe an elderly woman who developed dialysis-requiring severe kidney failure soon after a brief febrile illness and was found to have strongly positive myeloperoxidase ANCA. Contrary to expectation, her kidney biopsy revealed active acute tubulointerstitial nephritis without any necrotizing or crescentic glomerular lesions, even though her urine contained dysmorphic erythrocytes, raising the possibility that glomerular injury was present but not captured in the sampled tissue. She recovered steadily with corticosteroid therapy and became dialysis-independent. This case serves as a reminder that ANCA-associated kidney disease can present without its classic glomerular signature, and that an early biopsy followed by timely immunosuppression can salvage kidney function in this unusual phenotype.

## Introduction

Antineutrophil cytoplasmic antibody (ANCA)-associated vasculitis (AAV) is a pauci-immune small-vessel vasculitis that predominantly affects the kidneys through necrotizing and crescentic glomerulonephritis [1,2]. Although tubulointerstitial inflammation is frequently observed in renal biopsies from patients with AAV, it usually occurs in conjunction with glomerular injury and is considered a secondary manifestation of the disease [2,3].

Isolated acute tubulointerstitial nephritis (ATIN) without evidence of necrotizing or crescentic glomerulonephritis represents an uncommon renal phenotype of AAV [3,4]. This atypical presentation has been reported only in a limited number of cases, most commonly in association with myeloperoxidase (MPO)-ANCA positivity [3,5-7]. The underlying pathogenesis remains incompletely understood, although injury involving the peritubular capillaries has been proposed as a potential mechanism [3,4].

This phenotype differs from glomerular ANCA-associated vasculitis not in the severity of kidney failure, which may be just as profound, but in the urinary sediment and biopsy appearance. The sediment is comparatively bland - modest microscopic hematuria with only a small number of dysmorphic erythrocytes, an absence of red cell casts, no sterile pyuria, and proteinuria that remains in the subnephrotic range, in contrast to the florid nephritic sediment that accompanies crescentic disease. On biopsy, the glomeruli show no necrotizing lesions and no crescents, which is not the same as being entirely normal, since mild mesangial expansion and background sclerosis may still be present. It is this mismatch, an ANCA-positive patient whose biopsy resembles ordinary interstitial nephritis, that makes the diagnosis so easy to overlook [3,5]. Early identification and prompt initiation of immunosuppressive therapy may improve renal outcomes and potentially prevent progression to classical ANCA-associated crescentic glomerulonephritis [8-10].

We report a case of a 69-year-old woman who presented with severe kidney failure requiring dialysis, microscopic hematuria, subnephrotic proteinuria, and strongly positive MPO-ANCA. Kidney biopsy demonstrated active acute tubulointerstitial nephritis with acute tubular injury in the absence of necrotizing or crescentic glomerulonephritis. The patient showed significant recovery of renal function following corticosteroid therapy. We also reviewed the published literature and compared the clinical characteristics of our patient with previously reported cases of isolated ANCA-associated tubulointerstitial nephritis.

## Case presentation

A 69-year-old woman was referred to our tertiary care center after routine investigations performed by her primary care physician revealed markedly elevated serum creatinine. A total of 15 days before presentation, she had experienced a low-grade febrile illness for which she received empirical treatment for two to three days; the prescription could not be retrieved, and neither the patient nor her family was able to identify the drugs she had been given, so a temporal association with a nephrotoxic or hypersensitivity-inducing agent cannot be formally excluded. She was, however, questioned directly and on more than one occasion about the use of non-steroidal anti-inflammatory drugs, proton pump inhibitors, antibiotics taken on her own initiative, and any ayurvedic, homeopathic, or over-the-counter alternative preparation, and she consistently denied all of these. Following resolution of her fever, she developed progressive anorexia, nausea, fatigue, generalized weakness, and bilateral pedal edema, prompting further evaluation. She did not report documented weight loss or night sweats.

She had no previously diagnosed comorbidities and was taking no regular medication. There was no history of recent vaccination, no preceding diarrheal or urinary tract infection apart from the febrile episode described, and no personal or family history of tuberculosis, kidney disease, malignancy, or autoimmune disease. No earlier serum creatinine measurement was available from any source, so her true baseline kidney function is unknown.

On admission, the patient maintained an adequate urine output and was neither oliguric nor anuric at any stage of her illness. She denied symptoms suggestive of systemic vasculitis, including skin rash, arthralgia, hemoptysis, urticaria, cutaneous ulceration, visual disturbance, or peripheral neuropathy. She also had no dry eyes, dry mouth, parotid swelling, or arthritis to suggest Sjögren syndrome or another connective tissue disorder. Her blood pressure was 146/84 mmHg. Respiratory examination revealed fine bilateral bibasal crepitations, while the remainder of the systemic examination was unremarkable. A bedside urine dipstick performed as part of the systemic examination showed 2+ protein and 2+ blood. Initial laboratory investigations demonstrated advanced kidney failure with serum creatinine of 10.71 mg/dL and blood urea of 213 mg/dL. Additional investigations revealed anemia, leukocytosis, thrombocytosis, hyponatremia, and mild hyperkalemia (Table 1).

**Table 1:** Admission laboratory investigations. ANCA: antineutrophil cytoplasmic antibody; MPO: myeloperoxidase; PR3: proteinase 3; HPF: high-power field; ELISA: enzyme-linked immunosorbent assay

Parameters	Patient value	Reference range (adults)
Hemoglobin (g/dL)	7.6	12.0-15.5
Total leukocyte count (/mm³)	15,300	4,000-11,000
Platelet count (/mm³)	675,000	150,000-450,000
Blood urea (mg/dL)	213.0	15.0-40.0
Serum creatinine (mg/dL)	10.71	0.6-1.2
Sodium (mEq/L)	123.0	135.0-145.0
Potassium (mEq/L)	5.91	3.5-5.0
Complement C3 (mg/dL)	144.0	90.0-180.0
Complement C4 (mg/dL)	32.5	10.0-40.0
PR3-ANCA (U/mL)	1.10	<5.0
MPO-ANCA, ELISA (U/mL)	50.0	<5.0
MPO-ANCA, indirect immunofluorescence	+++	Negative
24-h urinary protein (g/24 h)	0.94	<0.15
Urinalysis (dipstick proteinuria)	+2	Negative
Microscopic hematuria (RBCs/HPF)	15.0	0-2.0
Dysmorphic erythrocytes (RBCs/HPF)	5.0	Absent
Red blood cell casts	None	Absent
White blood cell casts	None	Absent
Granular casts	None	Absent
Waxy casts	None	Absent

Routine urinalysis and microscopy were the next investigations performed before any serological testing was requested. Urinalysis showed +2 proteinuria and microscopic hematuria with approximately 15 red blood cells per high-power field, of which five per high-power field (approximately 30%) were dysmorphic. No red blood cell casts, white blood cell casts, granular casts, or waxy casts were identified on urine microscopy, and pyuria was absent. A total of 24-h urinary protein excretion was 0.94 g/day; urinary albumin was not quantified separately.

The finding of severe kidney failure of unknown etiology accompanied by an active urinary sediment prompted a second and specifically targeted line of investigation directed at an immunologically mediated cause; complement levels and ANCA serology were therefore requested because of the urine findings and were not part of a routine admission panel. Complement levels (C3 and C4) were within the normal range. Antinuclear antibody was negative, which, together with normal complement levels and the absence of sicca symptoms, argued against systemic lupus erythematosus and Sjögren syndrome. Serological evaluation demonstrated strongly positive myeloperoxidase (MPO)-ANCA by both enzyme-linked immunosorbent assay (ELISA) (50 U/mL) and indirect immunofluorescence (+++), while proteinase 3 (PR3)-ANCA was negative. Serum protein electrophoresis, serum free light-chain assay, and immunofixation electrophoresis were unremarkable.

Ultrasonography showed normal-sized kidneys with normal cortical echogenicity, maintained corticomedullary differentiation, and no hydronephrosis, findings that argued against long-standing chronic kidney disease and made a kidney biopsy both safe and informative. Given the combination of severe kidney failure, microscopic hematuria, subnephrotic-range proteinuria, normal-sized kidneys, and MPO-ANCA positivity, a kidney biopsy was performed. High-resolution computed tomography of the thorax was undertaken to evaluate for pulmonary involvement and demonstrated acute alveolar and interstitial edema without radiological features suggestive of pulmonary vasculitis. Pulmonary function testing showed a mild restrictive ventilatory defect.

Histopathological examination of the renal biopsy included two cores containing 10 glomeruli, two of which were globally sclerosed, leaving eight glomeruli available for assessment. Light microscopy demonstrated mild mesangial matrix expansion without evidence of necrotizing or crescentic glomerulonephritis; none of the assessable glomeruli showed fibrinoid necrosis, capillary loop rupture, endocapillary hypercellularity, or segmental sclerosis, and no arteritis or arteriolar fibrinoid necrosis was seen in the sampled vessels. Prominent lymphocytic tubulitis with active tubulointerstitial inflammation and acute tubular injury was present; the infiltrate was predominantly mononuclear, without a prominent eosinophilic component or granulomata, an appearance that, together with the absence of pyuria on urine microscopy, argues against, though does not entirely exclude, a drug-induced hypersensitivity reaction. Mild interstitial fibrosis and tubular atrophy involving approximately 15-20% of the sampled cortex, together with focal glomerulosclerosis (20%), were also identified. Immunofluorescence microscopy was negative, with no staining for immunoglobulins or complement components and no linear glomerular basement membrane staining, confirming a pauci-immune pattern. Electron microscopy was not performed. Light microscopy and immunofluorescence had already established pauci-immune tubulointerstitial disease in a patient with strongly positive MPO-ANCA, and ultrastructural examination would not have changed either the diagnosis or the treatment; we acknowledge, however, that this leaves subtle glomerular ultrastructural injury formally unexcluded. Overall, the biopsy was consistent with active acute tubulointerstitial nephritis with acute tubular injury in the absence of histopathological evidence of glomerular vasculitic lesions (Figures 1, 2).

**Figure 1:**
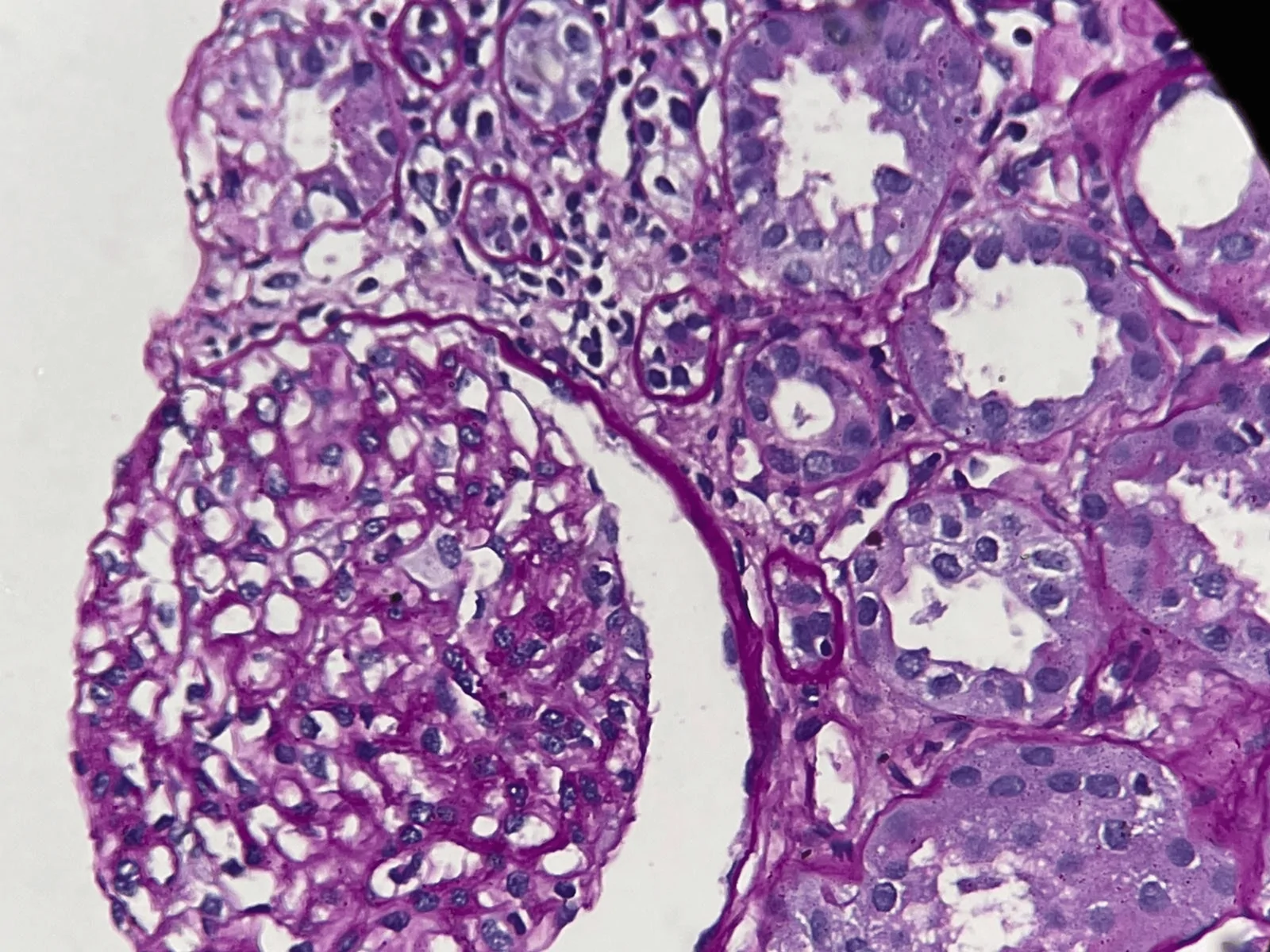
Periodic acid-Schiff (PAS) stain (×400) demonstrating preserved glomerular architecture without necrotizing or crescentic glomerulonephritis. Adjacent tubules show tubular injury with surrounding interstitial inflammatory infiltrates.

**Figure 2:**
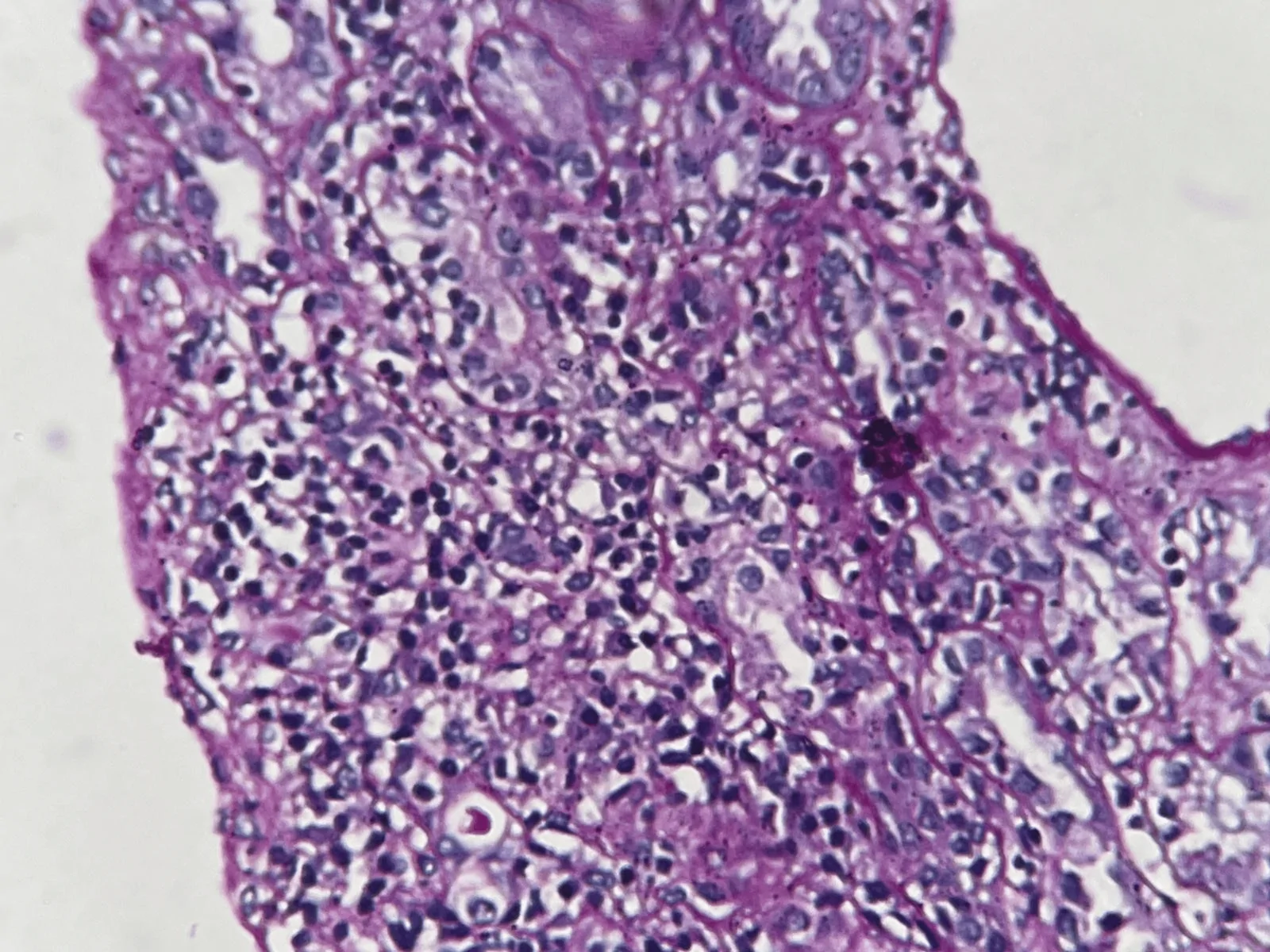
Periodic acid-Schiff (PAS) stain (×400) showing dense lymphocytic tubulointerstitial inflammatory infiltrates with tubulitis and acute tubular epithelial injury, consistent with active acute tubulointerstitial nephritis.

Because of advanced renal failure with uremic symptoms, the patient underwent three sessions of intermittent hemodialysis. All three sessions were given before the kidney biopsy, in order to correct uremia and hyperkalemia and to reduce the bleeding risk associated with a percutaneous biopsy in an advanced uremic state. The kidney biopsy was performed before any immunosuppression was started. Once the biopsy had demonstrated active tubulointerstitial nephritis with a pauci-immune pattern, and in the context of strongly positive MPO-ANCA with no alternative explanation, the illness was interpreted as kidney-limited ANCA-associated vasculitis and treated accordingly with intravenous methylprednisolone 500 mg daily for three consecutive days, followed by oral prednisolone at 1 mg/kg/day [8,9].

The patient demonstrated gradual clinical and biochemical improvement during hospitalization. She was discharged with a urine output of approximately 1.0-1.5 L/day and a serum creatinine of 6.35 mg/dL. A double-lumen hemodialysis catheter was retained in situ because of the possibility of requiring further dialysis.

During follow-up, renal function progressively improved, allowing discontinuation of hemodialysis. Serum creatinine decreased from 8.38 mg/dL at the first week of follow-up to 4.36 mg/dL at two weeks and 2.56 mg/dL at one month. Microscopic hematuria gradually resolved, urinary sediment became inactive, and corticosteroids were tapered according to clinical response (Table 2). MPO-ANCA was not repeated during follow-up; remission was judged instead on the basis of the progressive fall in serum creatinine, independence from dialysis, and resolution of hematuria and proteinuria, and we recognize this as a shortcoming addressed in the limitations below. Maintenance immunosuppression with azathioprine, mycophenolate, or rituximab was considered but intentionally deferred. The patient had achieved a complete clinical and biochemical response to corticosteroid therapy alone; therefore, the decision was made to continue a gradual prednisolone taper with close clinical and urinary monitoring. It was clearly understood that maintenance therapy would be initiated and a repeat biopsy considered if serum creatinine plateaued or increased, if the urinary sediment became active again, or if features of systemic vasculitis developed.

**Table 2:** Serial laboratory investigations and clinical course during follow-up. HPF: high-power field

Parameters	1-week follow-up	2-week follow-up	1-month follow-up	Reference range (adults)
Hemoglobin (g/dL)	13.7	12.0	10.7	Female: 12.0-15.5
Total leukocyte count (/mm³)	20,100	16,430	8,600	4,000-11,000
Platelet count (/mm³)	275,000	129,000	205,000	150,000-450,000
Blood urea (mg/dL)	419.0	241.0	81.0	15.0-40.0
Serum creatinine (mg/dL)	8.38	4.36	2.56	0.6-1.2
Proteinuria (dipstick)	+1	+1	Negative	Negative
Microscopic hematuria (RBCs/HPF)	3.0	8.0	Absent	0-2.0
Dysmorphic erythrocytes (RBCs/HPF)	Absent	6.0	Absent	Absent
RBC casts	None	None	None	Absent
WBC casts	None	None	None	Absent
Granular casts	None	None	None	Absent
Waxy casts	None	None	None	Absent
Dialysis	One hemodialysis session	Not required	Not required	-
Corticosteroid therapy	Prednisolone 1 mg/kg/day continued	Prednisolone 1 mg/kg/day continued	Prednisolone tapered by 10 mg	-
Dialysis catheter	Double-lumen catheter retained in situ	In situ	Removed	-

## Discussion

Clinical significance of the case

Renal involvement in ANCA-associated vasculitis (AAV) classically manifests as pauci-immune necrotizing crescentic glomerulonephritis. Although tubulointerstitial inflammation is a frequent accompanying histological finding, isolated acute tubulointerstitial nephritis (ATIN) without glomerular involvement remains an exceptionally uncommon presentation. Recognition of this entity is clinically important because patients may present with severe kidney failure despite the absence of the characteristic glomerular lesions traditionally associated with AAV.

Our patient presented with severe kidney failure, microscopic hematuria, subnephrotic-range proteinuria, and strongly positive MPO-ANCA serology. Despite a clinical presentation suggestive of ANCA-associated renal disease, kidney biopsy demonstrated active acute tubulointerstitial nephritis with acute tubular injury and no evidence of necrotizing or crescentic glomerulonephritis. The central lesson of this case is therefore the recognition of ANCA-associated kidney disease in a patient whose biopsy lacked its typical findings as follows: the diagnosis rested on the combination of strongly positive serology, an active urinary sediment, and a pauci-immune histological pattern, and clinicians should not allow the absence of crescentic glomerulonephritis on biopsy to exclude AAV from consideration.

Reconciling the urinary sediment with the biopsy findings

The central tension in this case is that the urine and the biopsy pointed in different directions. Approximately 30% of the erythrocytes in her sediment were dysmorphic, and she excreted 0.94 g of protein daily, both of which suggest a glomerular source of injury, and yet none of the eight assessable glomeruli showed necrotizing or crescentic change. There are three plausible explanations. The first, and in our view the most likely, is sampling error. Glomerular involvement in ANCA-associated vasculitis is characteristically focal and segmental, and a biopsy containing eight assessable glomeruli samples a vanishingly small fraction of the approximately one million glomeruli in each kidney, so a lesion affecting a minority of glomeruli can easily be missed. The second is that severe tubulointerstitial inflammation with tubulitis and disruption of tubular basement membranes can itself permit erythrocytes to enter the tubular lumen, where changes in osmolality and pH along the nephron deform them, generating dysmorphic cells in the absence of primary glomerular disease. The third is that a glomerular lesion was present but at a stage too early to be resolved by light microscopy, which electron microscopy might conceivably have detected.

For this reason, we prefer to describe our patient as having tubulointerstitial nephritis without histopathological evidence of glomerular involvement, rather than disease that genuinely spares the glomerulus, and we suggest that the same caveat applies to several previously reported cases [3-7]. This does not weaken the message of the case. If anything, it strengthens it, because it means that a biopsy showing only interstitial inflammation in an ANCA-positive patient should not be taken as reassurance, particularly when the sediment is nephritic. There is also a hopeful corollary. If the biopsy captured only uninvolved glomeruli, this may indicate that the disease was detected before crescents had formed. This would be consistent with the good functional recovery observed in our patient and in most reported cases and would support performing a biopsy at the earliest opportunity rather than waiting for a classical nephritic picture to develop.

Isolated tubulointerstitial disease is not unique to ANCA-associated vasculitis. A comparable pattern, in which interstitial inflammation dominates and glomerular involvement is minimal or absent, has been described in lupus nephritis, and there too the diagnosis has depended on biopsy rather than serology [11]. This parallel supports the view that the tubulointerstitium can be a primary target of systemic autoimmune injury rather than merely a bystander compartment.

The interpretation of the ANCA result

Because the diagnosis in this case rested heavily on serology, the performance of the assay deserves comment. Solid-phase immunoassays for MPO-ANCA have high specificity when applied to patients with a compatible clinical syndrome, but low-level positivity is well recognized in infection, tuberculosis, malignancy, other autoimmune disease, and with drugs such as hydralazine, propylthiouracil, minocycline and levamisole-adulterated cocaine, and the positive predictive value of any single result falls sharply when the test is ordered indiscriminately [12]. Several features argue against a false-positive result here. The ELISA titer was 10 times the upper reference limit, indirect immunofluorescence was concordantly and strongly positive, proteinase 3 (PR3)-ANCA was negative, and the serology was accompanied by an active urinary sediment and a compatible biopsy. The general principle nonetheless holds, and it is one reason we did not begin immunosuppression on the serological result alone - an isolated ANCA result should never by itself drive treatment.

Comparison with published literature

Only a limited number of cases of isolated ANCA-associated tubulointerstitial nephritis have been described in the literature (Table 3). Most reported patients have been elderly, predominantly MPO-ANCA positive, and have presented with non-specific constitutional symptoms accompanied by acute kidney injury. Histopathological examination has consistently demonstrated prominent tubulointerstitial inflammation without necrotizing or crescentic glomerular lesions.

**Table 3:** Published cases of ANCA-associated tubulointerstitial nephritis without glomerulonephritis. ANCA: antineutrophil cytoplasmic antibody; MPO: myeloperoxidase; PR3: proteinase 3; IIF: indirect immunofluorescence; IFTA: interstitial fibrosis and tubular atrophy

Studies	Patient	Clinical presentation	ANCA profile	Renal biopsy findings	Treatment	Outcome
He et al. (2022) [3]	Case 1 (78-year-old male)	Fever, abdominal distension, anorexia, oliguria, edema, dyspnea, hypertension	MPO-ANCA: 184 AU/mL	Interstitial edema and fibrosis, dense inflammatory infiltrates, tubular basement membrane thickening, tubular atrophy	Intravenous methylprednisolone followed by oral prednisolone and rituximab	Serum creatinine improved (481 to 223 μmol/L); ANCA became negative after 2 months
He et al. (2022) [3]	Case 2 (64-year-old male)	Fever, fatigue, myalgia, arthralgia, edema, generalized weakness, interstitial lung disease	MPO-ANCA: 239 AU/mL	Diffuse interstitial inflammatory infiltrates with tubular destruction	Oral prednisolone (1 mg/kg/day) and cyclophosphamide	Serum creatinine improved (159 to 86 μmol/L); ANCA became negative
He et al. (2022) [3]	Case 3 (67-year-old female)	Hypertension	MPO-ANCA: 39.4 AU/mL	Interstitial edema and fibrosis, inflammatory infiltrates, tubular basement membrane thickening, tubular atrophy, tubular IgA, κ-chain, and λ-chain staining	Oral prednisolone (1 mg/kg/day) and cyclophosphamide	Improvement in renal function; ANCA became negative after 4 months
Kim et al. (2021) [5]	70-year-old woman	Hypertension, intermittent fever, back pain, bilateral shoulder and leg pain, left foot drop	MPO-ANCA and PR3-ANCA positive	Segmental mesangial proliferation, focal tubular atrophy, mononuclear interstitial infiltration with fibrosis, diffuse podocyte foot process effacement	High-dose glucocorticoids followed by azathioprine	Clinical improvement with recovery of neurological symptoms
Hassani et al. (2013) [6]	75-year-old man	Acute kidney injury	MPO-ANCA positive (IIF 1:320; ELISA: 28.8 IU/mL)	Acute tubulointerstitial nephritis without glomerular involvement	Corticosteroids	Improvement in renal function; ANCA became negative
Guo et al. (2020) [7]	77-year-old woman	Hypertension and lethargy	PR3-ANCA 114 U/mL	Acute tubulointerstitial nephritis with interstitial granuloma and minor glomerular abnormalities	Corticosteroids	Relapse after 1 year
Present case	69-year-old woman	Acute kidney injury following febrile illness; anorexia, nausea, pedal edema; microscopic hematuria; subnephrotic proteinuria	MPO-ANCA 50 U/mL (ELISA); IIF +++; PR3-ANCA negative	Active acute tubulointerstitial nephritis with acute tubular injury; mild IFTA (15-20%); focal glomerulosclerosis (20%); no necrotizing or crescentic glomerulonephritis	Intravenous methylprednisolone (500 mg/day for 3 days) followed by oral prednisolone (1 mg/kg/day); temporary hemodialysis	Progressive recovery of renal function with serum creatinine decreasing from 10.71 mg/dL to 2.56 mg/dL at 1 month

The largest published case series by He et al. reported three patients with MPO-ANCA-associated isolated interstitial nephritis who showed significant improvement following immunosuppressive therapy [3]. Similar observations have been described by Kim et al., Hassani et al., and Guo et al., supporting the concept that isolated tubulointerstitial nephritis represents a rare but distinct renal phenotype within the spectrum of ANCA-associated vasculitis [5-7]. Reported patients generally demonstrated lower serum creatinine, less proteinuria, fewer urinary red blood cells, and lower MPO-ANCA titers than patients with classical crescentic glomerulonephritis [3,5,10].

The underlying pathophysiological mechanism remains uncertain. Previous studies have proposed that inflammation involving the peritubular capillaries may represent the primary site of vascular injury, resulting in tubulointerstitial inflammation without significant glomerular involvement [3,4]. Whether isolated ATIN represents an early stage of ANCA-associated renal disease or a distinct clinicopathological subtype remains unresolved because of the limited number of reported cases.

Comparison with institutional experience

We also compared our patient with our own cohort of ANCA-associated crescentic glomerulonephritis, and we present this comparison as local context rather than as evidence. That retrospective review was not designed to identify tubulointerstitial disease and contained no such case, so it cannot be used to support the existence of this phenotype; what it does offer is a reference point for what classical ANCA-associated kidney disease looks like in the same population. Against that reference, patients with crescentic glomerulonephritis showed substantially greater urinary abnormalities, including higher proteinuria and more pronounced microscopic hematuria than the present case, and a greater proportion presented with oliguria. Their mean serum creatinine was 7.07 mg/dL, mean hemoglobin was 8.4 g/dL, and mean MPO-ANCA titer was 48 U/mL. It is worth noting that the mean creatinine of the crescentic cohort was in fact lower than our patient's value of 10.71 mg/dL, so the difference between the two presentations lay in the urinary findings rather than in the severity of kidney failure. This cautions against assuming the tubulointerstitial phenotype is invariably milder, even though the reported cases, including that of He et al., have generally recovered well [3].

Implications for clinical practice

This case underscores several important clinical considerations. First, persistent MPO-ANCA positivity in a patient with unexplained kidney failure should prompt consideration of ANCA-associated vasculitis even when urinary findings are modest and systemic manifestations are absent. Second, kidney biopsy remains essential for distinguishing isolated tubulointerstitial nephritis from classical pauci-immune crescentic glomerulonephritis, as management strategies and prognosis may differ. Third, the biopsy and the treatment decision answered two different questions. Histology confirmed active tubulointerstitial nephritis and excluded an immune-complex or anti-glomerular basement membrane process, which justified immunosuppression; the choice of pulse methylprednisolone rather than oral corticosteroids alone rested on dialysis dependence and on guideline recommendations for organ-threatening ANCA-associated disease, not on any histological demonstration of vasculitis [8,9]. It should be acknowledged that the published cases provide no direct evidence for pulse corticosteroids in this specific phenotype. All reported patients received corticosteroids, intravenously in one case and orally in the remainder, with cyclophosphamide, azathioprine, or rituximab added in several, but none of these reports establishes a preferred regimen for isolated tubulointerstitial disease, and our approach was extrapolated from the management of severe ANCA-associated kidney disease in general.

An important observation from previous reports is that isolated tubulointerstitial nephritis may not always remain histologically stable. Wen and Chen described a patient whose initial biopsy demonstrated isolated tubulointerstitial nephritis but who subsequently developed necrotizing crescentic glomerulonephritis on repeat biopsy [10]. Although our patient experienced progressive renal recovery with corticosteroid therapy alone, this observation highlights the importance of careful long-term follow-up and consideration of repeat kidney biopsy in patients with persistent or worsening renal dysfunction.

Limitations

This case has several important limitations that should be considered when interpreting the findings. First, the most important limitation concerns the biopsy sample. The specimen contained 10 glomeruli, of which two were globally sclerosed, leaving eight glomeruli available for assessment. Although 10 glomeruli is conventionally regarded as an adequate biopsy sample, 20 or more glomeruli are considered ideal when a focal or segmental lesion is being sought. Therefore, in a patient presenting with severe kidney failure and dysmorphic hematuria, the possibility that focal necrotizing or crescentic lesions were not sampled cannot be excluded. This represents the principal caveat in describing the disease as isolated.

Second, electron microscopy was not performed for the reasons described above; therefore, subtle glomerular ultrastructural injury cannot be formally excluded. Third, MPO-ANCA was not repeated during follow-up, so serological remission could not be documented, and clinical and urinary parameters were used instead for follow-up assessment. Fourth, no baseline creatinine level was available, although normal kidney size and echotexture argue against significant pre-existing chronic kidney disease. Fifth, the drugs administered during the preceding febrile illness could not be identified, so a contributory drug-induced component cannot be entirely excluded, even though the histological findings and strongly positive serology favor ANCA-associated disease. Finally, this is a single case with only one month of documented follow-up, and longer observation will be required to determine whether this phenotype relapses or evolves into crescentic glomerulonephritis.

## Conclusions

Acute tubulointerstitial nephritis without histopathological evidence of glomerular involvement represents an uncommon renal manifestation of MPO-ANCA-associated vasculitis. In patients presenting with unexplained rapidly progressive kidney failure and MPO-ANCA positivity, ANCA-associated vasculitis should remain an important diagnostic consideration even in the absence of crescentic glomerulonephritis. Early kidney biopsy is critical for establishing the diagnosis and guiding appropriate immunosuppressive therapy. When the urinary sediment suggests glomerular bleeding but the biopsy does not, assume sampling error rather than exclude it; these patients warrant continued surveillance with a low threshold for maintenance immunosuppression and repeat biopsy. Prompt recognition and treatment may permit substantial recovery of renal function and potentially improve long-term renal outcomes.
